# Effects of changes in health insurance reimbursement level on outpatient service utilization of rural diabetics: evidence from Jiangsu Province, China

**DOI:** 10.1186/1472-6963-14-185

**Published:** 2014-04-23

**Authors:** Lu Zhang, Zhonghua Wang, Dongfu Qian, Jian Ni

**Affiliations:** 1 School of Health Policy & Management, Nanjing Medical University, Hanzhong Road 140, Nanjing 210029, Jiangsu Province, P.R China; 2 Carey Business School, Johns Hopkins University, 100 International Drive, Baltimore, MD 21202, USA

**Keywords:** Outpatient reimbursement, Health insurance, Health service utilization, Type 2 DM, China, Rural health

## Abstract

**Background:**

Outpatient reimbursement levels of the New Rural Cooperative Medical Scheme have changed in recent years in China, and those changes may have a greater impact on patients with chronic diseases due to their higher outpatient expenses. This study represents the first attempt to identify the effects of reimbursement level on outpatient service utilization for chronic patients in rural China and it also gives strong estimation results by conducting a tracer illness study in order to control for possible biases associated with studying several diseases together.

**Methods:**

This study used difference-in-differences models to examine how changes in yearly maximum reimbursement amount and outpatient reimbursement rates affected rural residents with type 2 diabetes in three counties in Jiangsu Province, China. Other factors, such as sex, age and severity of illness, were also included in the model estimations. To make sure the treated group and control group are comparable, Propensity Score Match (PSM) was used to analysis the gender, age and severity of illness of the two groups.

**Results:**

The results indicate that an increase in yearly maximum reimbursement amount for outpatient visits could cause an increase in yearly total outpatient expenses for patients with type 2 diabetes mellitus. However, changes in outpatient reimbursement rates between 2010 and 2011 did not significantly affect the utilization of different types of health institution.

**Conclusions:**

The reimbursement rates of village clinics should be substantially increased from the existing basis and the gap of reimbursement rates among different institutions should be further widened. It is also important for village clinics to improve their services. Moreover, measures to improve the quality of care and scope of services at lower-level healthcare institutions, and promote the health service utilization of rural women should be considered.

## Background

With an aging population and dramatic changes in social and economic conditions, China is experiencing an epidemic of chronic diseases. Chronic diseases now account for an estimated 80% of deaths and 70% of disability-adjusted life-years lost in China [1]. The direct and indirect economic costs of chronic diseases are already substantial and are likely to grow [2]. In the next 10 years, China is projected to lose $558 billion [3] due to chronic diseases. The total number of cases of chronic disease in 2008 was up to 260 million, including 160 million cases in rural areas [4]. Compared to urban patients, rural patients with relatively low incomes have significantly lower capacity to cope with chronic disease-related economic burden [5]. Compared with costs associated with acute diseases, outpatient expenses associated with chronic illnesses, such as diabetes, generally account for a bigger proportion of all medical expenses for patients. Patients with chronic diseases typically need drugs to control their illness rather than hospitalization. Thus, medical expenditures for most patients with chronic diseases are mainly outpatient expenses. Therefore, lightening the burden of outpatient expenses for chronic patients was one of the objectives of the New Rural Cooperative Medical Scheme (NRCMS).

Introduced in 2003, NRCMS was implemented in all rural counties in China by the end of 2008 [6]. In 2011, the number of rural residents participating in NRCMS was 832 million, representing a participation rate of over 97% [7]. NRCMS is run by the government and financed in part through flat-rate household contributions (the poor and certain groups have their contributions subsidized) and in part through central and local government subsidies [8]. It operates at the county level and may vary in design and implementation across counties. Specific NRCMS policies in each county may be made by local health authorities according to local conditions [9]. At present, immediate reimbursement for medical care has been realized in most rural areas in China, and farmers who participate in NRCMS usually only pay out-of-pocket medical fees (self-paid medical fees). Health institutions will bill the government (the local office of NRCMS) directly for those medical expenses that can be reimbursed by NRCMS. Rural patients pay out-of-pocket for their medical treatment beyond the scope of coverage including beyond the local fixed reimbursement rate or yearly maximum reimbursement amounts. At present, both outpatient expenses and inpatient expenses for people covered by NRCMS may be reimbursed in most counties [10]. However, the bulk of NRCMS reimbursement is for inpatient costs in order to prevent catastrophic healthcare expenditure. The proportion of risk-pooling funds for outpatient reimbursement is small and accounts for 30% or less of overall total insurance pooling funds. For outpatients, the level of reimbursement for chronic diseases is the same as for acute diseases in spite of the generally heavier burden of outpatient expenses for people with chronic diseases [11].

Annual funding for NRCMS has increased in recent years. Reimbursement level (including outpatient reimbursement level) has also changed. Those changes may have a greater impact on patients with chronic diseases due to their higher outpatient expenses, but few studies have examined the impact on outpatient service utilization and expenses. This study seeks to quantitatively analyze the impact of changes in outpatient reimbursement level on outpatient service utilization and expenses.

Health service utilization is affected by many factors, which can be divided into two main classes. The first class is patients’ characteristics including sex, age, and income. For example, in a study about the National Health Insurance in Taiwan, patients who had lower educational attainment and income levels were less likely to use outpatient services [12]. The second class is environmental or external factors including medical insurance level and how health services are organized and delivered. There are already some studies about the effects of medical insurance or reimbursement level. Some have shown that having health insurance may increase health service utilization. For example, the community-based health insurance in Rwanda was found to be associated with significantly increased utilization of health services [13]. Similarly, Kondo and Shigeoka [14] have found that expansion of universal health insurance coverage in Japan resulted in large increases in hospital admissions, inpatient days and out-patient visits to hospitals. Speculating on the relationship between health insurance status and utilization, Wagstaff and Lindelow have argued that health insurance allows people more chances to use advanced technology and high value-added medical services [15]. In research carried out in Taiwan, National Health Insurance was believed to increase medical service utilization by lowering the financial barrier for insured older patients [16]. In addition, there are also some studies about how the changes in insurance copayment or coinsurance rate might affect health service utilization. For example, several studies have separately analyzed the effects of the changes of cost-sharing policies on the utilization of clinical preventive services [17], the use of inhaled medications in older patients with chronic obstructive pulmonary disease or asthma [18] and the emergency hospitalizations and physician visits [19]. However, to the best of our knowledge, there are no studies that analyze the impact of outpatient reimbursement level changes of NRCMS on chronic patients’ outpatient service utilization in China.

In China, most of the literature on NRCMS reimbursement level focuses on Risk-pooling of Outpatient Service, Family Account Service or allotment of funds between outpatient and inpatient care [20-22]. Only a few studies have focused on the impact of outpatient reimbursement level on the behaviours of patients of chronic diseases. For example, a study about the nature and characteristics of outpatient reimbursement level of chronic diseases under NRCMS was carried out in 32 counties in China [23]; and the effects of NRCMS outpatient co-ordination plan on residents’ choices of healthcare institutions were examined by Huang et al. [24]. However, these researchers used only cross-sectional data and their analysis tended to be descriptive in nature. Also, their findings require cautious interpretation because the data were collected at a single point in time. Moreover, there are no studies that investigate the impact of outpatient reimbursement level on utilization in China by controlling for possible biases associated with lumping diseases together. Some studies have argued that disease-related variables are more important than socio-demographic factors in predicting use of different kinds of provider [25,26].

In this study, two years of panel data were used to quantitatively analyze the effects of outpatient reimbursement rate changes and yearly maximum reimbursement amounts for outpatient visits on outpatient service utilization and corresponding medical expense of rural patients with type 2 diabetes mellitus (DM) in order to provide empirical evidence for improving outpatient reimbursement level for rural patients with chronic diseases in Jiangsu Province. The reimbursement rate in this research is the policy reimbursement rate. In addition, the policy reimbursement rate and actual reimbursement rate of the majority of outpatients is equal because the research subjects were typical rural patients with type 2 DM, which is in very different from the health conditions of inpatients.

## Hypotheses

In China, the per capita income of most rural residents is still relatively low. Therefore, changes in outpatient reimbursement level are more likely to affect rural patients’ ability to pay and may have an effect on the choice and utilization of different types of rural health institution. Theoretically, yearly maximum reimbursement amount has a positive impact on total outpatient medical expenditure, and the change of reimbursement rate at every level of health institution has a positive impact on corresponding utilization at this type of health institution. Thus, we make the following hypotheses:

H_1_: Increase of the yearly maximum reimbursement amount for outpatient services will increase yearly total outpatient expenses of rural patients with type 2 DM.

H_2_: Increase of outpatient reimbursement rates at every level of health institution will increase the outpatient service utilization at these institutions by rural patients with type 2 DM; for example, if the outpatient reimbursement rate of a certain level of medical institution is high enough, more patients will choose to access health services there.

The study subjects were rural patients with type 2 DM. In China, the number of diabetic patients has been increasing substantially [27]. It has been estimated that in 2007/2008, among adults 20 years of age or older, there were 92.4 million persons with diabetes, 43.1 million of whom resided in rural areas [28]. Type 2 diabetic patients account for about 95% of all diabetics in China [29]. Diabetes generates a heavy economic, social and familial burden and its complications are equally serious [30,31]. Rural patients with lower income have less capacity to pay for health care than their urban counterparts [32]. Because type 2 DM is a disease that can be treated by many healthcare providers, it is commonly regarded as a tracer illness that could be used to identify factors that influence the utilization of outpatient services at different types of health care institution.

In the next section, we briefly describe the data, followed by a discussion of the methods used. Results from the analysis and a discussion about relevant issues and policy implications are then presented. The final section provides a conclusion to this paper.

## Methods

### Data collection

In Jiangsu Province, a province-level NRCMS management database was established in 2009, from which data were extracted for this study. Using a random cluster sampling method, we selected three counties: Gaoyou, Pukou, and Rudong. Located in the middle of Jiangsu Province, these three counties are in the mid range of economic development in the province. Data on 14,169 type 2 diabetic outpatients in the three counties with complete data for two years were extracted from the database according to ICD-10 code. For patients to be included in the study, they had to: (1) have type 2 DM and (2) be age 10 years and over (Individuals aged 9 years and under rarely have type 2 DM and their information is usually not complete). The exclusion criteria were: (1) those with severe diabetic complications (e.g., diabetic foot or severe diabetic retinopathy) and (2) with terminal illness (e.g., cancer, AIDS) or other medical problems that could affect the comparisons of outcomes. Our data covered related visits to three levels of health institutions in the three counties in 2010 and 2011.

Generally speaking, health care in rural China is delivered by healthcare institutions at the village, township and county levels. Village-level clinics are usually perceived to provide the lowest standard of care at the lowest price, whereas county hospitals are perceived to provide the highest standard of care, but at the highest price. Township health centers (THCs), at the secondary level, have full-time doctors providing primary health care and supervising public health and medical care services provided by village clinics. Village clinics or health stations, staffed by one or more part-time health workers, provide basic health care. In 2010, the proportions of visits to village clinics, THCs and county-level hospitals were 40.7%, 37.1% and 22.2%, respectively, and the proportions of visit in 2011 were 39.7%, 39.4% and 20.9% in Jiangsu province, respectively. The National Essential Drugs Policy (NEDP) came into effect at THCs and village clinics by the mid-2010. Under the NEDP, all grass-roots health institutions below the county level follow a policy of zero mark-up of retail drug prices above cost [33].

In the three sampled counties, NEDP was simultaneously implemented at THCs and village clinics. Therefore, the three counties have the same NEDP implementation history, which makes the present analysis possible. But there are also some differences between the three counties in relation to reimbursement level. In Gaoyou County, the outpatient reimbursement rate was raised to 35% at village clinics and to 30% at THCs from 2010 to 2011, and there was no change at county-level hospitals. The yearly maximum reimbursement amount for outpatient visits increased by 100 yuan (or RMB) (about US$ 15.8) from 2010 to 2011. In Pukou County, there was no change between 2010 and 2011 in reimbursement rates and the yearly maximum reimbursement amount. In Rudong County, the outpatient reimbursement rates at village clinics, THCs, and county-level hospitals were increased to 30%, 25% and 20%, respectively, from 2010 to 2011. The yearly maximum reimbursement amount for outpatient visits was raised by 40 yuan from 2010 to 2011. The specific outpatient reimbursement level in three counties is shown in Table 1.

**Table 1:** Outpatient reimbursement policy in three sample counties

**Items**	**2010**			**2011**		
	**Gaoyou**	**Pukou**	**Rudong**	**Gaoyou**	**Pukou**	**Rudong**
Outpatient reimbursement rate(%)						
Village clinics	25	40	25	35	40	30
THCs	25	30	20	30	30	25
County-level hospital	20	20	15	20	20	20
Yearly maximum payment amount	500	600	460	600	600	500
Rural per capita net income (yuan RMB)	8825	11188	9120	10170	13204	10500

Additionally, there are several reasons for the validity of model estimation in this study. Firstly, the research subjects were typical rural patients with type 2 DM. There is no obvious difference in health service quality for these patients, such as treatment and drugs prescribed among different sample counties. According to interviews with administrative staff members of NRCMS, patients without serious complications are less likely to seek health service outside their counties. Secondly, patients only can be reimbursed at the local cooperative medical management center, thus patients from other counties were not recorded in the database. Thirdly, usually only patients with serious complications will go to higher level health institutions outside their own counties to seek treatment. In fact, there are very few such patients. In addition, those patients should be excluded from our research data due to their serious complications.

### Data analysis

In order to analyze the effects of outpatient reimbursement level changes between 2010 and 2011, difference-in-differences (DID) with matching was used to make model estimates. Each record in this data reflects the nature and numbers of outpatient utilization of every level of health institution in each year. In other words, it is not the record of each visit by outpatients. Thus discrete choice models can not be used for our data. Moreover,the objective of this study is to examine whether the change of outpatient reimbursement rates at every level of health institution can impact on the ratio of utilization of corresponding health institution. Therefore,we used DID models, which are also suitable for our panel data. A quasi-experimental technique used in econometrics, DID measures the effect of an intervention or treatment at a given period in time. In a DID analysis, study objects are divided into two groups and observed for two time periods. One group is exposed to an intervention in the second period but not in the first period. The second group is not exposed to an intervention during either period. In the case where the same units within a group are observed in each time period, the average gain in the control group is subtracted from the average gain in the intervention group. This removes biases in the second period comparisons between the intervention and control groups that could be the result of permanent differences between those groups, as well as biases from comparisons over time in the intervention group that could be the result of trends. With two year panel data, the model for a generic member of groups can be expressed as:

(1) $$Y=\beta_{0}+\beta_{1}\mathrm{dB}+\delta_{0}d_{2}+\delta_{1}d_{2}\;*\;\mathrm{dB}+U$$

Where Y is the outcome of interest, d_2_ is a dummy variable for the second time period. The dummy variable dB captures possible differences between the intervention and control groups prior to the reimbursement level change. The time period dummy, d_2_, captures aggregate factors that would cause changes in Y, even in the absence of a reimbursement level change. The coefficient of interest, δ_1_, multiplies the interaction term, d_2_*dB, which is the same as a dummy variable equal to one for those observations in the intervention group in the second period. The DID estimate is

(2) $$\Delta \delta_{1}=\left({\bar{Y}}_{b,2^{-\;}}{\bar{Y}}_{b,1}\right)-\left({\bar{Y}}_{a,2^{-}\;}{\bar{Y}}_{a,1}\right)$$

Inference based on even moderate sample sizes in each of the four groups is straightforward, and is easily made robust to different group/time period variances in the regression framework.

DID models have become a widely used method to investigate changes in policy or legislation [34]. There are a few recent studies using DID to measure the effects of changes in health policy. For example, DID was used to estimate the effects of National Health Insurance on utilization in Taiwan [19]. DID was also used to assess the effects of implementing an outpatient dialysis global budget on different types of utilization for hemodialysis patients [35].

Of the three counties, outpatients from a county where there was no change in yearly maximum reimbursement amount for outpatient visits and reimbursement rates belonged to a control group. In the DID model, any bias caused by variables common to the three counties are implicitly controlled for, even when these variables are unobserved.

The following equations were employed to estimate the effects of changes in reimbursement level on utilization for type 2 diabetic patients. Equation 3 was employed to estimate the effects of changes in yearly maximum reimbursement amount for outpatient visits on yearly total outpatient expenses.

(3) $$Y=\beta_{0}+\beta_{1}T+\beta_{2}P_{1}+\beta_{3}T\;*\;P_{1}+\beta_{4}X+U$$

Dependent variable Y represents yearly total outpatient expenditures of patients; and because it is a skewed distribution, the logarithm value of yearly total outpatient expenses was used in model estimations. T is a dummy variable indicating the year when the policy was implemented. P_1_ is a dummy variable indicating the intervention status of yearly maximum reimbursement amount. T*P_1_ tells us how yearly maximum reimbursement amount changes impose different impact between different years.

Equation 4 was employed to estimate the effect of changes in outpatient reimbursement rates on outpatient visits at different healthcare institutions.

(4) $$\begin{array}{ll} Y_{i}= & \beta_{0}+\beta_{1}T+\beta_{2}P_{i}+\beta_{3}T\;*\;P_{i}+\beta_{4}X+U\\ & \left(i=1,2,3\right) \end{array}$$

Dependent variables Y_1_, Y_2_ and Y_3_ represent the ratios of visits by patients to village clinics, THCs and county-level hospitals, which reflect the likelihood of using these health institutions. Specifically, Y_i_ = (number of visits to one type of health institution by a patient during one year)/(number of total visits to all health institutions of a patient during one year) (i = 1, 2, 3). T*P_i_ tells us how reimbursement level changes impose different impacts between different years. T is a dummy variable indicating the year when the policy was implemented. P_i_ is a dummy variable indicating the intervention status of the county’s outpatient reimbursement rates.

In the above equations, X is the background covariates including age, sex and severity of illness. In order to address potential differences in health service utilization among different patients, the study used six age groups: 10 ~ 17 years, 18 ~ 30 years, 31 ~ 45 years, 46 ~ 60 years, 61 ~ 80 years, and 81 years and above. The severity of illness was divided into three categories: low severity, medium severity and high severity. The groupings were suggested by expert panels composed of physicians and researchers, and were based on different drug categories and dosages during a one-year period. Specifically, the high severity category covers patients using medicines that treated severe diabetes, like Neutral Insulin Injection, Somatostatin for Injection, Pancreatic Kininogenase for Injection, and requires that these drugs’ subtotal dosage was more than half of all diabetic drugs’ dosage for this patient during a 1-year period. The low severity group refers to those who often used the first and second generation of sulfonylureas or biguanides drugs, and requires that these drugs’ subtotal dosage was more than half of all diabetic drugs’ dosage for this patient during a 1-year period. The rest are in the medium severity group, who mainly used medicine that treats moderate diabetes like Glipizide Controlled-release Tablets, Glibenclamide Tablets, etc. U indicates all unobserved factors affecting patients’ behaviors like patients’ income and education. These variables were not included in the NRCMS management database. Detailed description of the variables is displayed in Table 2.

**Table 2:** Description of variables

**Variable**	**Description**	**Mean(S.D.)**			
		**Total**	**Village clinic**	**THC**	**County-level hospital**
Dependent variables					
Year	=1 if Year = 2010; =0 otherwise.	0.38(0.49)	0.39(0.49)	0.38(0.49)	0.40(0.49)
Sex	=1 if Sex = female; =0 otherwise.	0.43(0.50)	0.41(0.49)	0.43(0.49)	0.39(0.48)
Age					
Age 1^**^	=1 if 18 ≤ Age ≤ 30; =0 otherwise.	0.02(0.15)	0.02(0.13)	0.02(0.15)	0.02(0.14)
Age 2	=1 if 10 ≤ Age ≤ 17; =0 otherwise.	0.02(0.14)	0.02(0.12)	0.02(0.15)	0.01(0.10)
Age 3	=1 if 31 ≤ Age ≤ 45; =0 otherwise.	0.11(0.31)	0.08(0.28)	0.11(0.31)	0.11(0.31)
Age 4	=1 if 46 ≤ Age ≤ 60; =0 otherwise.	0.34(0.47)	0.32(0.47)	0.32(0.47)	0.35(0.48)
Age 5	=1 if 61 ≤ Age ≤ 80; =0 otherwise.	0.44(0.50)	0.48(0.50)	0.45(0.50)	0.45(0.50)
Age 6	=1 if 81 ≤ Age; =0 otherwise	0.06(0.24)	0.08(0.24)	0.06(0.24)	0.05(0.23)
Severity of illness					
Low severity^**^	=1 if low severity; =0 otherwise.	0.27(0.44)	0.24(0.43)	0.29(0.45)	0.12(0.32)
Medium severity	=1 if medium severity; =0 otherwise.	0.40(0.50)	0.42(0.49)	0.40(0.49)	0.30(0.46)
High severity	=1 if high severity; =0 otherwise.	0.33(0.50)	0.34(0.47)	0.31(0.46)	0.58(0.49)
Diff-rate^*^	=1 if different = 5 or different = 10; =0 otherwise.		0.87(0.33)	0.83(0.37)	0.74(0.43)
Diff-amount	The difference of the annual maximum outpatient payment amount between 2010 and 2011				
Diff-amount 1^**^	=1 if different = 0; =0 otherwise.	0.21(0.41)			
Diff-amount 2	=1 if different = 40; =0 otherwise.	0.54(0.50)			
Diff-amount 3	=1 if different = 100; =0 otherwise.	0.25(0.43)			
Independent variables					
Totalfee	yearly total outpatient fee	344.08(310.05)			
Ln (Totalfee)	=Ln(yearly total outpatient fee)	5.47(0.86)			
Rate	Rate of patients’ outpatient visit number at different types of health institutions to the total yearly outpatient visit number		0.8(0.28)	0.75(0.33)	0.64(0.35)

Note: * means the difference of outpatient reimbursement rate between 2010 and 2011; ** means omitted group.

## Results

In order to make sure the treated group and control group are comparable, Propensity Score Match (PSM) was used to analysis the gender, age and severity of illness of the two groups. The treated group and control group were not the same when we analyzed different level institutions; for example, at village clinics, the reimbursement rate was not changed in Pukou, so we regarded outpatients of Pukou as control group, while at county-level hospitals, the reimbursement rates of Gaoyou and Pukou were both unchanged. So when we analyzed the health service utilization of county-level hospitals, we considered Gaoyou and Pukou as the control group. Thus, we did PSM three times for three different institution models. After performing PSM, the standard biases of gender, age and severity of illness are all reduced, which means the characteristics of gender, age and severity of illness of the two groups become closer, and the two groups become more comparable. Because of the limitation of length, we only report the result of PSM in the THC model in Table 3. However, because the policy variable was divided into three groups for the estimation model of yearly total outpatient expenses and PSM is not suitable for three groups, we did not use PSM before DID estimation about yearly total outpatient expenses.

**Table 3:** The results of Propensity Score Match of THCs

**Variable**		**Mean treated**	**Mean control**	**Bias%**	**Reductive bias%**	**T-value**	**P-value**
Sex	Matched	1.576	1.549	5.4		3.07	0.002
	Unmatched	1.560	1.560	0.0	100.0	−0.00	1.000
Age 2	Matched	0.019	0.048	−16.2		−10.53	0.000
	Unmatched	0.020	0.020	0.0	100.0	−0.00	1.000
Age 3	Matched	0.112	0.119	−2.1		−1.19	0.235
	Unmatched	0.120	0.120	0.0	100.0	−0.00	1.000
Age 4	Matched	0.326	0.346	−4.4		−2.44	0.015
	Unmatched	0.332	0.332	0.0	100.0	−0.00	1.000
Age 5	Matched	0.454	0.435	3.8		2.10	0.036
	Unmatched	0.445	0.445	0.0	100.0	0.00	1.000
Age6	Matched	0.070	0.014	28.2		13.00	0.000
	Unmatched	0.062	0.062	0.0	100.0	0.00	1.000
Severity of illness							
Medium severity	Matched	0.394	0.391	0.6		0.34	0.736
	Unmatched	0.399	0.399	0.0	100.0	0.00	1.000
High severity	Matched	0.372	0.412	−30.0		−17.56	0.000
	unmatched	0.247	0.247	0.0	100.0	0.00	1.000

Table 4 shows the effects of changes in yearly maximum reimbursement amount for outpatient visits on yearly total outpatient expenses, which were estimated according to the equation (3). The annual per capita outpatient expenses in 2010 and 2011 were 491.0 and 456.5 yuan, respectively. According to the results of estimation, sex, yearly maximum reimbursement amount, and severity of illness achieved statistical significance. As for sex, female patients’ yearly total outpatient expenses were 2.6% lower than those of male patients. In addition, the yearly total expenses of patients with medium severity and high severity were obviously higher than those of patients with low severity. These results are in line with the situation in Jiangsu province where the yearly total outpatient expenses of individuals with type 2 DM increased sharply with increasing severity of illness. The influence of changes in yearly maximum reimbursement amount was significant at the 0.01 level. Compared with the patients whose maximum reimbursement amount remained unchanged, an increase in total outpatient expenses was 9.1% more likely for the patients whose yearly maximum reimbursement amount increased by 40 yuan and 17.0% more likely for the patients whose yearly maximum reimbursement amount increased by 100 yuan. The results provide empirical support for Hypothesis 1.

**Table 4:** Results of effect on yearly total outpatient medical fees

**Variable**	**Yearly total outpatient medical fees**		
	**Coefficient**	**Exp(coefficient)-1**	**t-value**
Severity of illness			
Medium severity	1.210***	2.065	68.02
High severity	2.439***	10.462	138.33
Sex	−0.026**	−0.026	−2.12
Age 2	−0.005	−0.005	−0.07
Age 3	0.023	0.023	0.69
Age 4	0.001	0.001	0.08
Age 5	0.03	0.030	0.57
Age 6	0.021	0.021	0.35
year	−0.013	−0.013	−0.58
Diff-amount 2	0.115***	0.122	5.80
Diff-amount 3	−0.159***	0.147	−6.70
Diff-amount 2 *year	0.087***	0.091	3.13
Diff-amount 3 *year	0.157***	0.170	4.71

Note: * Indicates significance at 0.1; ** Implies significance at 0.05; *** Indicates significance at 0.01.

Table 5 shows the effects of changes in outpatient reimbursement rates on the ratio of using different types of healthcare institution, which were estimated according to the equation (4). For village clinics, THCs and county-level hospitals, the corresponding models were estimated accordingly. The coefficients of THCs and county-level hospitals were found to be statistically significant. According to the results of models, compared with the control group, outpatients of the treated group are 5.99% more likely to visit THCs and 2.34% more likely to visit county-level hospitals. In addition, possible interactions between age, sex, and severity of illness variables were also examined.

**Table 5:** Results of effect on the ratio of visits by outpatients to different types of health care provider

**Variable**	**Ratio of visits by outpatients to different health institutions**					
	**Village clinic**		**THC**		**County-level hospital**	
	**Coefficient**	**t-value**	**Coefficient**	**t-value**	**Coefficient**	**t-value**
year	−1.720	−1.30	−2.744***	−2.91	−2.480***	−2.6
Diff-rate	−8.253***	−7.32	−19.993***	−20.86	−33.381***	−36.29
Diff-rate *year	−0.434	−0.29	5.985***	4.55	2.341***	1.97
Sex	−4.040***	−6.48	−2.670***	−3.84	0.978**	1.66
Age 2	4.496***	2.04	6.622***	3.30	−4.444	−1.55
Age 3	−2.293	−1.40	1.243	0.73	−3.567***	−1.99
Age 4	−0.214	−0.14	−6.626***	−4.13	−7.902***	−4.67
Age 5	2.769**	1.87	−9.225***	−5.79	−10.736***	−6.03
Age 6	11.031***	6.67	−6.371***	−3.29	−9.905***	−4.79
Severity of illness						
Medium severity	−12.493***	−31.18	4.011***	5.32	5.114***	5.26
High severity	−32.349***	−64.49	6.071***	7.99	19.221***	30.59

Note: * Indicates significance at 0.1; ** Implies significance at 0.05; *** Indicates significance at 0.01.

According to the estimation model for village clinics, sex and three age categories were significant. The results indicate that, compared to male patients, female patients were 4.04% less likely to visit village clinics (which was statistically significant). Compared with the 18–30 age group, the 10–17 age group was 4.50% more likely to visit village clinics (which was statistically significant). As for patients in the other age groups, the older the patient, the higher was his/her likelihood of visiting a village clinic. For example, patients in the 61–80 and 80+ age groups were 2.77%, and 11.03%, respectively, more likely to visit village clinics than those in the 18–30 age group. The coefficients for severity of illness were negative and statistically significant. Patients with medium severity were 12.49% less likely to visit village clinics than those in the low severity category. Similarly, patients with high severity were 32.35% less likely to visit village clinics than low-severity patients.

According to the estimation model for THCs, sex, severity of illness and four age categories were statistically significant. Specifically, females with type 2 DM were 2.67% less likely to visit THCs than their male counterparts. Compared with patients in the 18–30 age group, those in the 46–60, 61–80 and 80+ age groups were 6.63%, 9.23%and 6.37%, respectively, less likely to visit THCs. However, those in the 10-17age group were 6.62% more likely to visit THCs, and the other age groups did not differ significantly. Relative to those in the low severity category, patients with medium and high severity were 4.01% and 6.07%, respectively, more likely to visit THCs.

According to the estimation model for county-level hospitals, sex, severity of illness and four age categories were statistically significant. Specifically, females with type 2 DM were 0.99% more likely to visit THCs than their male counterparts. Compared with patients in the 18–30 age group, those in the 31–45,46-60, 61–80, and 80+ age groups were 3.57%, 7.90%, 10.74% and 9.91%, respectively, less likely to visit county-level hospitals. However, the other age groups did not differ significantly. Relative to the low severity category, patients with medium and high severity were 5.11% and 19.22%, respectively, more likely to visit county-level hospitals.

Contrary to the prediction of Hypothesis 2, outpatient reimbursement rate changes at village clinics, THCs and county-level hospitals did have a significant effect on the ratio of utilization. Possible reasons for this will be discussed in the next section.

## Discussions

This study examined the effects of outpatient reimbursement level on yearly total outpatient expenses and the ratio of using different types of healthcare institution by type 2 diabetic patients through DID model estimations, using two years of panel data. This represents the first attempt to identify the effects of reimbursement level on outpatient service utilization for chronic patients in rural China and it also gives strong estimation results by conducting a tracer illness study in order to control for possible biases associated with studying several diseases together. In this study, some other important variables were missing such as income, education, etc., which lead to estimation bias. However, all outpatients with type 2 DM in sample counties were included in this research and the two years of panel data of patients were used to compare, and these missing variables did not change between the two years. Therefore, this bias should be small and could be acceptable.

Generally, chronic diseases are characterized by long duration, complex etiology, frequent recurrent attacks, and difficulty to cure. On the other hand, many chronic diseases, such as diabetes, do not require expensive inpatient services. Most chronic situations can be managed by outpatient services [36]. But if individuals with chronic diseases do not receive timely outpatient services, many may incur higher health care costs due to late-stage utilization of inpatient services. In implementing the NRCMS, it is, therefore, important to ensure that patients with chronic diseases receive timely outpatient services by providing proper incentives such as outpatient reimbursements.

The research results provide support for Hypothesis 1 and show that an increase of yearly maximum reimbursement amount for outpatient visits can increase yearly total outpatient expenses. With an increase in reimbursement amount, the yearly total outpatient expenses rose rapidly. Relative to those patients whose reimbursement amount remained unchanged, the yearly total outpatient expenses of patients whose reimbursement amount increased by 100 yuan were 7.9% higher than those of patients whose reimbursement amount increased by only 40 yuan. According to the original data, there is no change in the price and kind of inspection and types of drugs used by sample patients between 2010 and 2011, the increase in outpatient expenditure mainly resulted from the increase of outpatient inspections and drugs utilization. However, the reasonability of the increase in utilization is still needed to further study in the future. It has been shown that low utilization of health services in rural China is often due to the heavy economic burden of seeking treatment for diseases [4]. Hence, increase in yearly maximum reimbursement amount for outpatient visits can increase outpatient service utilization and benefit individuals with chronic diseases such as type 2 DM. At the same time, with increased utilization, yearly total outpatient expenses will also rise correspondingly. Generally speaking, patients with chronic diseases suffer a bigger proportion of the burden of outpatient medical expenses than other patients. Therefore, some special outpatient reimbursement policies for rural patients with chronic diseases should be developed such as higher yearly maximum outpatient reimbursement amounts for chronic patients than other patients.

Contrary to the predictions of Hypothesis 2, apart from village clinics, changes in outpatient reimbursement rates of THCs and county-level hospitals between the two years did have a statistically significant effect on the ratio of visits to different types of healthcare institution. There might be three reasons for this situation. Firstly, the average medical expense per outpatient with type 2 DM of village clinics was relatively lower than that of THCs and county-level hospitals. Thus, the relatively smaller change in reimbursement relative to patients’ total out-of-pocket expenses and the smaller difference in fees charged in village clinics did not have a significant influence on ratio of utilization of health care provider. In the three counties examined, the largest difference of the reimbursement rate of a village clinic between two years was 10% (Table 1). In terms of actual out-of-pocket expenses after outpatient reimbursements have been taken into account, differences in expenses by patients in village clinics are even smaller. For example, in Gaoyou County, the average medical expense per outpatient at village clinics, THCs and county-level hospitals in 2010 and 2011 were 49.19 and 47.8 yuan, respectively; and the corresponding reimbursement rates were 25% and 35%, respectively. Thus, the average after-reimbursement out-of-pocket expenses per patient were 36.89 and 31.07 yuan, respectively. In other words, the difference in out-of-pocket expenses in village clinics between two years was only 5.82 yuan (Table 6). Therefore, it is possible that changes in outpatient reimbursement rates were too small to produce an economic incentive to guide patients to visit village clinics. Secondly, the quality of health services at THCs and county-level hospitals is much higher than that of village clinics. The change of outpatient reimbursement rate from low to high at THCs and county-level hospitals gives outpatients a chance to enjoy high-quality services. Some potential outpatients of THCs and county-level hospitals who were impeded by the high price of health service in the past might change their choice to receive health service at THCs and county-level hospitals after the change of outpatient reimbursement rate. Finally, the increase of yearly maximum reimbursement amount for outpatient visits can induce more patients to visit at the THCs and county-level hospitals, which may offset the attraction from the increase of outpatient reimbursement rates of village clinics. In short, all of the reasons mentioned above may contribute to the results of the model of effect on the ratio of utilization of different types of health care provider. Only if reimbursement rates are substantially increased at the village clinic level would village clinics become more attractive to patients. Needless to say, there is also a need for village clinics to improve their services. Patients typically take into consideration not just cost, but also quality and efficacy of care.

**Table 6:** Average out-of-pocket expense per outpatient with type 2 diabetes (yuan RMB)

**Items**		**Rudong**	**Gaoyou**	**Pukou**
Village Clinic	2010	40.87	49.19	64.46
	2011	35.90	47.8	55.80
	Change	−12.16%	−2.83%	−13.45%
THC	2010	78.12	72.91	105.91
	2011	69.23	70.21	85.25
	Change	−11.38%	−3.70%	−19.51%
County-level hospital	2010	211.63	166.26	160.68
	2011	186.25	193.84	152.30
	Change	−11.99%	16.59%	−5.22%

After controlling for other factors, our analysis shows that age did not have a statistically significant effect on yearly total outpatient expenses. Drug expenses accounted for more than 85% of yearly total outpatient expenses in 2010 and 2011 and there were no observable differences in terms of category and dosage of diabetic drugs used by the various age groups. Compared with those aged 18–30, patients in younger and older age groups were more likely to visit village clinics. There could be a simple explanation for this finding: 18–30 are much more likely to be migrant workers working in counties rather than staying behind in villages (like children and the elderly). They chose the institution because it’s more easily accessible. However, this should be further investigated and tested in future studies.

In addition, severity of illness affects the ratio of utilization of different types of healthcare institution. Patients in the high and medium severity categories were less likely to visit clinics than those with low severity. Patients with high severity were more likely to visit county-level hospitals, probably because they preferred to be treated at healthcare institutions that could handle more severe or complicated cases. Of the three levels of health institution in rural China, village clinics are usually seen as providing a relatively low standard of care but at the lowest price, whereas county hospitals are considered to have the highest standard of care but are more pricey. THCs, in the middle, have full-time doctors providing basic public health and medical care. Village clinics are typically staffed by one or more part-time primary healthcare workers who may not have the ability to deal with more serious illnesses. Thus, measures to improve the quality of care and scope of services at lower-level healthcare institutions, such as strengthening training and skills, better facilities and equipment, and more support and monitoring by higher-level institutions, should be taken if the goal is to avoid patients overwhelming county hospitals and to locate care closer to where rural people reside.

Our analysis shows that female patients were significantly less likely to visit village clinics and THCs, and significantly more likely to visit county-level hospitals. We speculate that rural women’s lower income and cultural level are reasons behind their lower utilization of services at clinics and THCs. It is generally known that women in rural China tend to have lower educational attainment than men and some of them are even illiterate. They may not recognize early symptoms of diabetes or pay little attention to early treatment until the disease becomes more serious. By that time, they may have no choice but to seek treatment at higher level hospitals due to disease exacerbation. Thus, with a view to furthering health equity, it is suggested that more attention be paid to health education, disease prevention and early detection among rural women. Special diabetes-related health promotion drives targeting rural women should be considered.

This study has a few limitations. As it is based on a secondary analysis of administrative data from a health insurance database, the analysis is limited by what the database contains. For instance, the lack of information about household income, other health problems and family or social support networks prevents us from conducting a more comprehensive analysis. We also did not examine diabetes-related inpatient expenses because two years of data are deemed insufficient to assess the impact of changes in outpatient reimbursement level on expenses related to hospital inpatient care. In addition, Outpatient reimbursement rates were different at every level of health institution and also different at every sample county. Thus, outpatient reimbursement rates can not be made as policy dummies for model estimation on outpatient medical expenditure. At the same time, Yearly maximum reimbursement amount was a total ceiling mount including reimbursable expenses at three levels of health institutions. Therefore, yearly maximum reimbursement amount can not be made as policy dummies for model estimation on the ratio of visits to every level of health institution. These may result in some bias due to information loss. These limitations need to be further study in the future.

## Conclusions

This study provides a quantitative description of the impact of changes in outpatient reimbursement level on outpatient service utilization and expenses of type 2 diabetic outpatients. The results showed that an increase in yearly maximum reimbursement amount for outpatient visits under NRCMS can increase outpatient service utilization and benefit individuals with chronic diseases such as type 2 DM. However, the increase by 5% or 10% in outpatient reimbursement rate in village clinics in sample counties was too slight to increase health service utilization of these clinics. In order to make village clinics more attractive and guide patients to visit them, their reimbursement rates should be substantially increased from the existing basis and the gap of reimbursement rates among different institutions should be further widened. Of course, it is also important for village clinics to improve their services. Moreover, measures to improve the quality of care and scope of services at lower-level healthcare institutions, and promote the health service utilization of rural women should be considered.

## Competing interests

The authors declare that they have no competing interests.

## Authors’ contributions

DQ led data collection, was involved in data analysis and took the lead on manuscript development. LZ was involved in data collection, analysis and drafted the manuscript. ZW gave statistical method supports. JN was involved in the initiation, design of the study and language editing. All authors have read and approved the entire manuscript.

## Pre-publication history

The pre-publication history for this paper can be accessed here:

http://www.biomedcentral.com/1472-6963/14/185/prepub
